# Artificial intelligence for pre-operative lymph node staging in colorectal cancer: a systematic review and meta-analysis

**DOI:** 10.1186/s12885-021-08773-w

**Published:** 2021-09-26

**Authors:** Sergei Bedrikovetski, Nagendra N. Dudi-Venkata, Hidde M. Kroon, Warren Seow, Ryash Vather, Gustavo Carneiro, James W. Moore, Tarik Sammour

**Affiliations:** 1 Discipline of Surgery, Faculty of Health and Medical Sciences, School of Medicine, University of Adelaide, Adelaide, South Australia Australia; 2 Department of Surgery, Colorectal Unit, Royal Adelaide Hospital, Adelaide, South Australia Australia; 3 Australian Institute for Machine Learning, School of Computer Science, University of Adelaide, Adelaide, South Australia Australia

**Keywords:** Colorectal cancer, Artificial intelligence, Radiomics, Deep learning, Machine learning, meta-analysis, Lymph node metastasis

## Abstract

**Background:**

Artificial intelligence (AI) is increasingly being used in medical imaging analysis. We aimed to evaluate the diagnostic accuracy of AI models used for detection of lymph node metastasis on pre-operative staging imaging for colorectal cancer.

**Methods:**

A systematic review was conducted according to PRISMA guidelines using a literature search of PubMed (MEDLINE), EMBASE, IEEE Xplore and the Cochrane Library for studies published from January 2010 to October 2020. Studies reporting on the accuracy of radiomics models and/or deep learning for the detection of lymph node metastasis in colorectal cancer by CT/MRI were included. Conference abstracts and studies reporting accuracy of image segmentation rather than nodal classification were excluded. The quality of the studies was assessed using a modified questionnaire of the QUADAS-2 criteria. Characteristics and diagnostic measures from each study were extracted. Pooling of area under the receiver operating characteristic curve (AUROC) was calculated in a meta-analysis.

**Results:**

Seventeen eligible studies were identified for inclusion in the systematic review, of which 12 used radiomics models and five used deep learning models. High risk of bias was found in two studies and there was significant heterogeneity among radiomics papers (73.0%). In rectal cancer, there was a per-patient AUROC of 0.808 (0.739–0.876) and 0.917 (0.882–0.952) for radiomics and deep learning models, respectively. Both models performed better than the radiologists who had an AUROC of 0.688 (0.603 to 0.772). Similarly in colorectal cancer, radiomics models with a per-patient AUROC of 0.727 (0.633–0.821) outperformed the radiologist who had an AUROC of 0.676 (0.627–0.725).

**Conclusion:**

AI models have the potential to predict lymph node metastasis more accurately in rectal and colorectal cancer, however, radiomics studies are heterogeneous and deep learning studies are scarce.

**Trial registration:**

PROSPERO CRD42020218004.

**Supplementary Information:**

The online version contains supplementary material available at 10.1186/s12885-021-08773-w.

## Background

Colorectal cancer (CRC) is the second most common malignancy and the third leading cause of cancer-related mortality in the world, accounting for 862,000 deaths annually [1]. CRC nodal metastases play a pivotal role in disease-free survival and in determining appropriate adjuvant and neoadjuvant treatment [2]. As a result of the application of preoperative staging MRI in patients with rectal cancer, neoadjuvant chemoradiation has become the standard of care in locally advanced tumours, resulting in improved local control and resectability. Owing to the lower accuracy of lymph node staging in colon cancer at diagnosis, neoadjuvant treatment is not as commonly recommended [3, 4]. However, this may change following the results of the recent Fluoropyrimidine, Oxaliplatin and Targeted Receptor Pre-Operative Therapy (FOXTROT) trial showing the safety and efficacy of neoadjuvant chemotherapy in patients with locally advanced colon cancer [5]. Therefore, improved accuracy in clinical nodal staging at diagnosis may become critical in surgical planning and targeting effective neoadjuvant treatment for these patients [6, 7].

Clinical staging of CRC is typically performed by radiologists assessing contrast enhanced computer tomography (CT) images in patients with colorectal cancer, and in addition, magnetic resonance imaging (MRI) in patients with rectal cancer. The staging accuracy of CT and MRI is affected by multiple factors, such as equipment performance, standardised imaging protocols, the reporting radiologist’s experience, and patient-specific factors. Overall, published series have reported a 70% accuracy of diagnosing lymph node metastasis on CT, and 69% on MRI using standard criteria [8, 9].

Current staging paradigms with its limited diagnostic and staging accuracy may be able to overcome by using Artificial intelligence (AI) models. AI-enabled radiomics involves the extraction of a large number of investigator defined features from medical images using advanced computational algorithms [10]. While radiomics models have been used to predict lymph node metastasis in CRC with partial success, previous studies by Ding et al. and Wang et al. demonstrate that deep learning algorithms have the potential to identify more subtle patterns that may elude conventional radiological and statistical methods [11–13]. Deep learning is a technique that involves the use of convolutional neural networks to self-educate an algorithm based on useful representations of images, thus bypassing the step of extracting manually designed features [14]. In recent years, radiomics nomograms and deep learning models have started to make a meaningful contribution to radiological diagnoses [15].

The aim of this systematic review and meta-analysis is to evaluate the accuracy of AI models in diagnosing lymph node metastasis on CT and/or MRI in colorectal cancer patients.

## Methods

### Search strategy

This systematic review and meta-analysis was performed according to the recommendations of the Preferred Reporting Items for Systematic Review and Meta-Analyses (PRISMA) guidelines and was registered with the International Prospective Register of Systematic Reviews with an analysis plan prior to conducting the research. A systematic search of the Cochrane Library, PubMed (MEDLINE), EMBASE and IEEE Xplore databases was performed for studies published between January 1st 2010 and October 1st 2020. The following search terms were used: artificial intelligence, deep learning, convolutional neural network, machine learning, automatic detection, radiomics, radiomic, CT/MRI, lymph node, lymph node metastasis, colon, rectal, colorectal (Additional file 1: Table S1). Reference lists of articles retrieved were also searched manually to identify additional eligible studies.

#### Study selection

Articles were included if they met the following criteria: (1) included patients with histopathological diagnosis of CRC; (2) developed or used a radiomics or deep learning algorithm to assess CT or MRI pre-operative lymph node metastasis detection and (3) published in English language. Exclusion criteria were (1) case reports, review articles, editorials, letters, comments, and conference abstracts; (2) studies focusing on segmentation or feature extraction methods only and (3) animal studies. After removing duplicates, titles and abstracts were reviewed for eligibility by two independent reviewers (SB and NNDV) using Covidence systematic review software (Veritas Health Innovation, Melbourne, Australia, available at www.covidence.org). Any disagreements were resolved by consensus arbitrated by a third author (TS).

#### Data extraction

Data from selected full-text articles were reviewed for reporting on the type of radiomics or deep learning model, study characteristics and outcome measures. The extracted data included the first author, year of publication, country, study type, number of patients, sample size for diagnostic accuracy, age, imaging modality, type of malignancy, AI model, and referenced standard. Data related to the accuracy of the radiologists’ assessment derived from studies using clinical nodal staging or clinical nomograms solely based on N-staging was also collected. To obtain diagnostic accuracy data of AI models and radiologists’ assessment, two-by-two contingency tables, sensitivity, specificity, accuracy, and area under the receiver operating characteristic curve (AUROC) were extracted or reconstructed. The primary endpoint was AUROC, secondary endpoints included sensitivity, specificity, and accuracy.

#### Quality assessment and publication Bias

The modified version as proposed by Sollini et al. of the Quality Assessment of Diagnostic Accuracy Studies (QUADAS-2) tool was used to access the methodological quality of the included studies [16]. Minimum criteria for fulfilling each QUADAS-2 item were discussed by two reviewers (SB and NNDV) and disagreements were resolved by consensus. Publication bias was assessed using the Egger regression test and is presented as a funnel plot of diagnostic AUROC.

#### Statistical analysis

Meta-analysis was performed using testing set results of studies that presented absolute numbers for AUROC and 95% confidence intervals, contingency tables or provided sufficient information to derive the numbers manually. If results were not reported in an independent test set, cross validation or full test sample results are presented in this review. When results of different AI algorithms were reported in one article, the proposed algorithm with the highest diagnostic performance was analysed.

Three software packages, MedCalc for Windows, version 16.4.3 (MedCalc Software, Ostend, Belgium), RevMan, version 5.3.21 and Meta-DiSc version 1.4, were utilised for statistical analysis. Missing data were computed using confusion matrix calculator or manually derived using formulas in Additional file 1: Table S2. Pooling sensitivity, specificity and AUROC data was conducted using the Mantel-Haenszel method (fixed-effects model) and the DerSimonian Laird method (random-effects model) [17, 18]. To assess heterogeneity between studies, the inconsistency index (I^2^) was used [19]. Heterogeneity was quantified as low, moderate, and high, with upper limits of 25, 50 and 75% for I^2^, respectively. Forrest plots were drawn to show AUROC estimates in each study in relation to the summary pooled estimate. A funnel plot was constructed to visually assess publication bias.

## Results

### Study selection

**Fig. 1:**
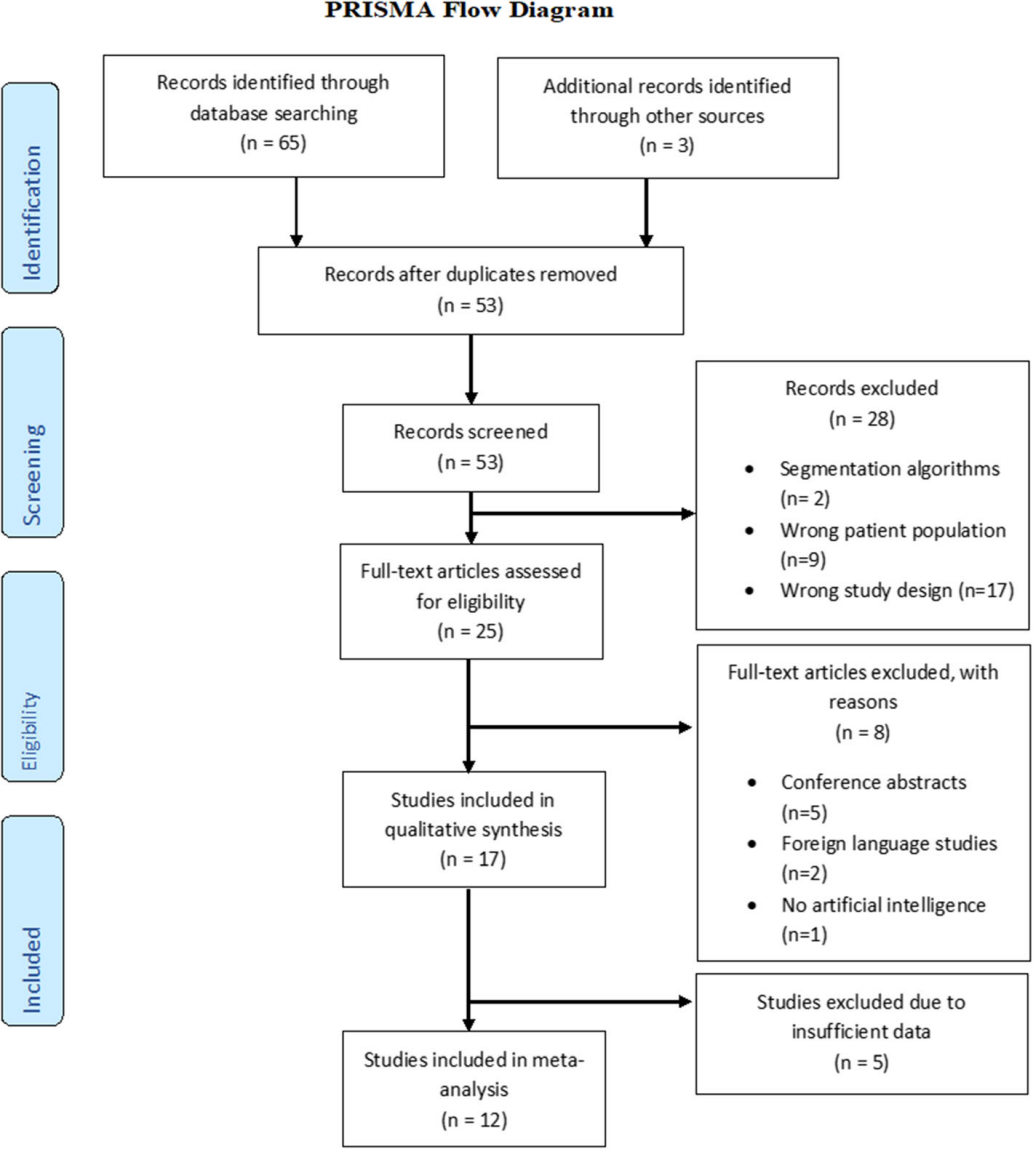
PRISMA flow chart outlining the selection of studies for review

A total of 68 studies were identified and 53 remained after removing duplicates. Review of titles and abstracts left 25 studies for full-text review. Finally, 17 studies were included in the systematic review, 12 of which could be used in the meta-analysis and five studies were excluded due to insufficient information (Fig. 1) [11, 12, 20–34].

### Study characteristics

Twelve studies used radiomics models and five used deep learning models (Additional file 1: Table S3). All included studies were published between 2011 and 2020. Study design was retrospective in 11 and prospective in six studies. Fourteen studies were single-center and three were multi-center. Patients were predominantly male with a median age of 60 years (54–64). Eight studies used MRI and nine used CT to train their algorithm. The type of malignancy was colorectal in three studies, colon only in two, and rectal only in 12. Eleven studies used per-patient diagnostic output (the patient is node positive or negative) and 6 studies used per-nodal diagnostic output of lymph node metastasis (each individual node analysed separately). Fifteen studies used the postoperative pathology report as reference standard, and one study used a radiology report as the reference standard. The reference standard for the one remaining study was not reported.

### Quality assessment and publication Bias

**Fig. 2:**
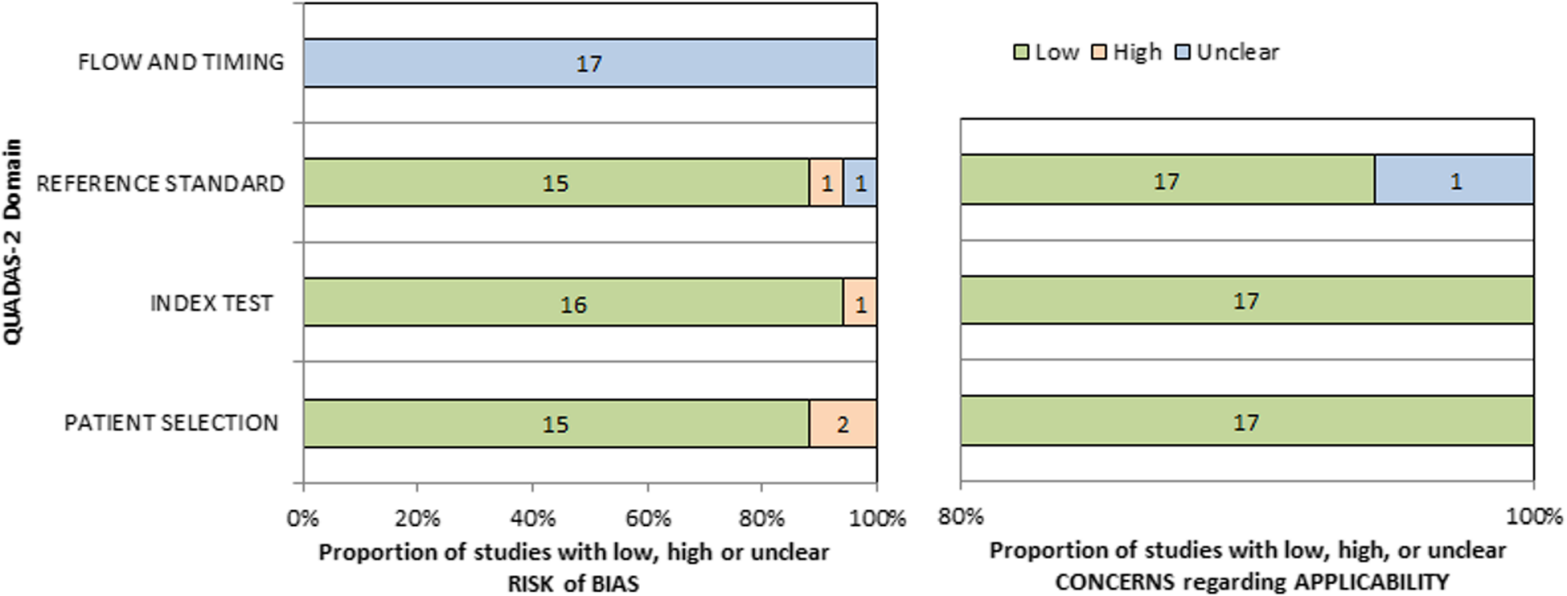
Summary of QUADAS-2 assessments of included studies

The methodologic quality of included studies is summarized in Fig. 2. As per the QUADAS-2 tool, risk of bias in patient selection was low in 15 (88%) studies and high in two (12%) studies. Risk of bias in the index test was high in one study (6%) and low in 16 (94%). Risk of bias in the reference standard test was low in 15 (88%), high in one study (6%) and unclear in one study (6%). Flow and timing had all 17 studies with unclear risk of bias. Overall applicability concerns were low (Additional file 1: Table S4). Funnel plot assessment (Additional file 1: Figure S1) showed no significant publication bias (Egger’s intercept 1.11, 95%CI − 1.22 to 3.42, *p* = 0.313).

### Diagnostic accuracy

**Fig. 3:**
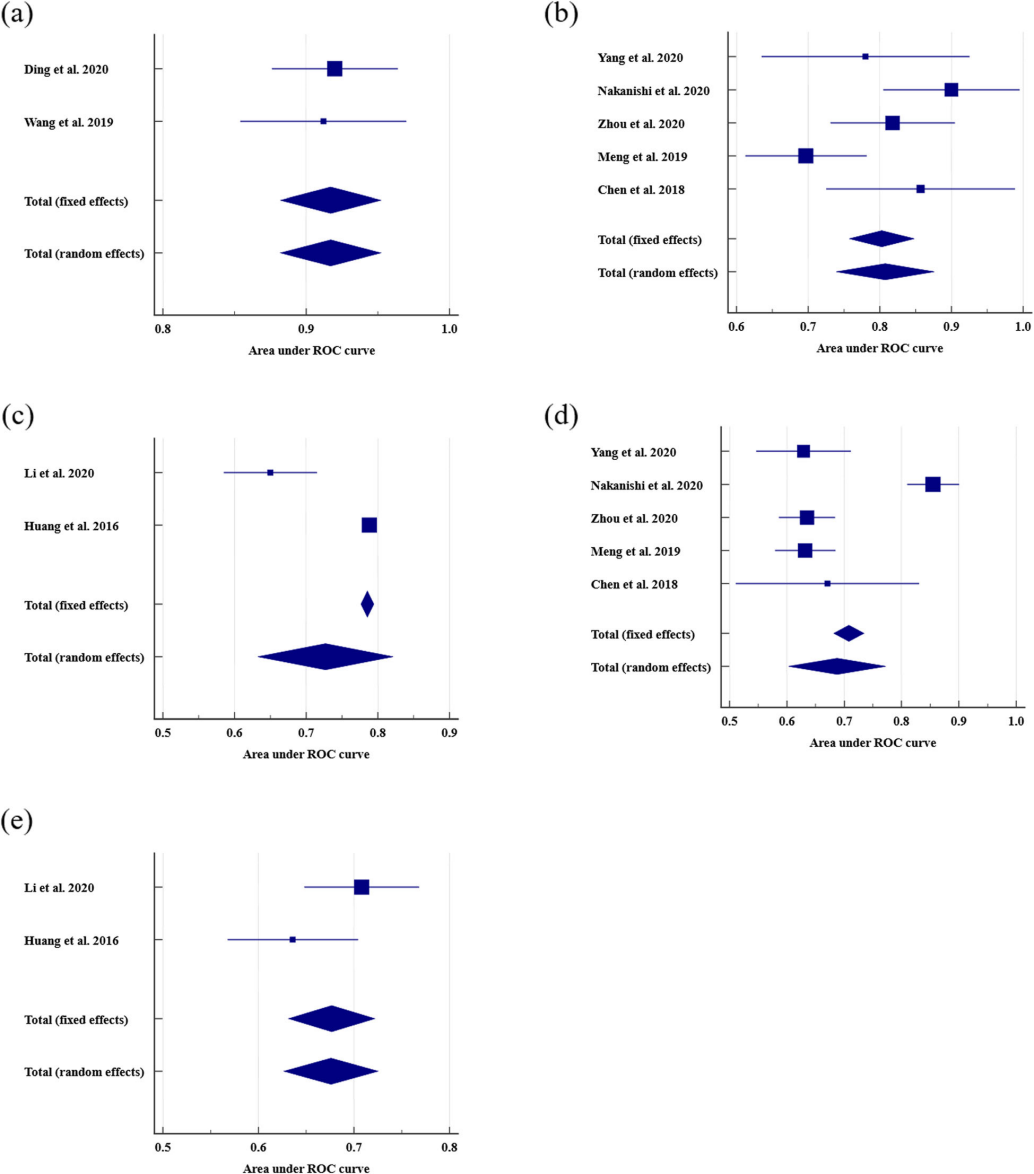
Forest plots of per-patient area under the receiver operating characteristic curve (AUROC). (**a**) Deep learning in rectal cancer, (**b**) radiomics in rectal cancer, (**c**) radiomics in colorectal cancer, (**d**) radiologist in rectal cancer and (**e**) radiologist in colorectal cancer

For the 12 studies that could be included in the quantitative analysis, 10 used radiomics and two used deep learning. For each outcome, summary estimates of sensitivity, specificity and AUROC were produced with 95% confidence intervals on a per-patient and per-nodal basis (Table 1). Pooled colorectal and rectal, per-patient and per-node detailed diagnostic measures reported by individual studies are shown in Table 2. The data for radiomics models in rectal cancer showed high heterogeneity with the exception of per-node AUROC and sensitivity. On a per-patient basis, radiomics in rectal cancer pooled AUROC was 0.808 (95%CI 0.739–0.876; Fig. 3) and pooled sensitivity and specificity were 0.776 (95%CI 0.685–0.851) and 0.676 (95%CI 0.608–0.739), respectively. On a per-nodal basis radiomics in rectal cancer pooled AUROC was 0.846 (95%CI 0.803–0.890) and pooled sensitivity and specificity were 0.896 (95%CI 0.834–0.941) and 0.743 (95%CI 0.665–0.811), respectively. On a per-patient basis radiomics in CRC pooled AUROC was 0.727 (95%CI 0.633–0.821). The radiologist per-patient assessment in rectal cancer pooled AUROC was 0.688 (95%CI 0.603 to 0.772), sensitivity was 0.678 (95%CI 0.628–0.726) and specificity was 0.701 (95%CI 0.667–0.733). Further, the radiologists per-patient assessment in CRC pooled AUROC was 0.676 (95%CI 0.627–0.725), sensitivity was 0.641 (95%CI 0.577–0.702) and specificity was 0.657 (95%CI 0.597–0.713). The deep learning data demonstrated low heterogeneity (I^2^ = 0.00%, *p* = 0.829), and on a per-patient basis, deep learning models outperformed radiomics and radiologist assessment in rectal cancer with an AUROC of 0.917 (95%CI 0.882–0.952). Deep learning sensitivity and specificity were reported in a single study as 0.889 and 0.935, respectively (Table 1).

**Table 1:** Results for deep learning radiomics models and radiologist in accuracy to detect lymph node metastasis

First author	TP	FP	TN	FN	PPV, %	NPV, %	Sensitivity, %	Specificity, %	Accuracy, %	AUROC	95%CI	Standard Error ^c^
**Deep learning**												
**Per-patient**												
Ding [12]	–	–	–	–	–	–	–	–	–	0.920	0.876–0.964	0.0224
Wang [11]	40	4	58	5	90.9	92.1	88.9	93.5	91.6	0.912 ^c^	0.842–0.958	0.0296
Glaser [25] ^a^	–	–	–	–	–	–	–	–	–	0.860	–	–
**Per-node**												
Lu [28]	–	–	–	–	–	–	–	–	–	0.912	–	–
Li [29]	–	–	–	–	–	–	–	–	94.4	–	–	–
**Radiomics**												
**Per-patient**												
Eresen [20]	29 ^c^	6 ^c^	33 ^c^	10 ^c^	82.8 ^c^	76.7 ^c^	74.36	84.62	79.49	0.825	0.778–0.872	0.0240
Li [21]	69 ^c^	44 ^c^	128 ^c^	67 ^c^	61.06	65.64	50.74	74.42	63.96	0.650	0.583–0.713	0.0331
Yang [22]	13 ^c^	5 ^c^	21 ^c^	2 ^c^	73.2 ^c^	90.5 ^c^	85.0	82.0	83.0	0.780	0.630–0.920	0.0740
Nakanishi [23]	–	–	–	–	–	–	–	–	–	0.900	0.800–0.990	0.0485
Zhou [24]	24 ^c^	27 ^c^	74 ^c^	5 ^c^	47.1	93.7	82.8	73.3	75.4	0.818	0.731–0.905	0.0444
Meng [26]	46 ^c^	36 ^c^	47 ^c^	17 ^c^	56.1 ^c^	73.4 ^c^	73.0	56.6	63.7	0.697	0.612–0.781	0.0431
Chen [30]	–	–	–	–	–	–	–	–	–	0.857	0.726–0.989	0.0671
Huang [31]	–	–	–	–	–	–	–	–	–	0.788	0.779–0.797	0.0046
**Per-node**												
Zhu [27]	18 ^c^	21 ^c^	32 ^c^	1 ^c^	46.2	97.0	94.7	60.4	69.4 ^c^	0.812	0.703–0.895	0.0490
Cai [32]	–	–	–	–	–	–	89	82	88	–	–	–
Tse [34]	–	–	–	–	–	–	–	–	91.0	–	–	–
Cui [33]	111 ^c^	17 ^c^	78 ^c^	14 ^c^	86.7 ^c^	85.0 ^c^	89	82	88	0.855 ^c^	0.801–0.898 ^c^	0.0247
**Radiologist**												
**Per-patient**												
Li [21] ^d^	94 ^c^	63 ^c^	109 ^c^	42 ^c^	59.9	72.2	69.1	63.4	65.9	0.708	0.645–0.765	0.0306
Eresen [20]	33 ^c^	23 ^c^	16 ^c^	6 ^c^	58.9 ^c^	72.7 ^c^	84.6	41.0	62.8	0.772	0.718–0.825	0.0273
Yang [22]	41	41	43	14	50.0	75.4	74.6	51.2	60.4	0.629 ^c^	0.543–0.709 ^c^	0.0423
Nakanishi [23] ^b^	71	0	147	29	100.0	83.5	71.0	100.0	88.3 ^c^	0.855 ^c^	0.805–0.896 ^c^	0.0232
Zhou [24] ^b^	49	89	215	38	35.5	85.0	56.3	70.7	67.5	0.635	0.585–0.683	0.0250
Meng [26] ^b^	88 ^c^	96 ^c^	124 ^c^	37 ^c^	47.8 ^c^	77.0 ^c^	55.9	70.4	61.3	0.632 ^c^	0.578–0.683 ^c^	0.0268
Chen [30]	–	–	–	–	–	–	–	–	–	0.671	0.511–0.831	0.0816
Huang [31]	58 ^c^	30 ^c^	69 ^c^	43 ^c^	65.9 ^c^	61.6 ^c^	57.4 ^c^	69.7 ^c^	63.5 ^c^	0.636 ^c^	0.565–0.702 ^c^	0.0349
**Per-node**												
Cui [33]	39 ^c^	101 ^c^	52 ^c^	36 ^c^	27.7 ^c^	59.1 ^c^	52 ^c^	34 ^c^	39.9 ^c^	0.430 ^c^	0.365–0.497 ^c^	0.0337

^a^ Values extracted from training set

^b^ Values extracted from total cohort

^c^ Manually derived/reconstructed values using formulas from Additional file 1: Table S2

^d^ Values extracted from clinical models

FN, false negative; FP, false positive; TN, true negative and TP, true positive; PPV, positive predictive value; NPV, negative predictive value; AUROC, area under the receiver operating characteristic; CI, confidence interval

**Table 2:** Pooled results of per-patient and per-node diagnosis from deep learning, radiomics and radiologists

Variable	Studies analysed	Type of malignancy	No. of studies	Pooled results (95% CI)	Heterogeneity (I^2^, %)	Heterogeneity *P* Value
**Deep learning**						
AUROC per-patient	[11, 12]	Rectal	2	0.917 (0.882–0.952)	0.00	0.829
**Radiomics**						
Sensitivity per-patient	[22, 24, 26]	Rectal	3	0.776 (0.685–0.851)	0.00	0.368
Sensitivity per-node	[27, 33]	Rectal	2	0.896 (0.834–0.941)	0.00	0.393
Specificity per-patient	[22, 24, 26]	Rectal	3	0.676 (0.608–0.739)	75.4	0.017
Specificity per-node	[27, 33]	Rectal	2	0.743 (0.665–0.811)	87.8	0.004
AUROC per patient	[21, 31]	Colorectal	2	0.727 (0.633–0.821)	94.1	< 0.0001
AUROC per patient	[22–24, 26, 30]	Rectal	5	0.808 (0.739–0.876)	63.3	0.028
AUROC per node	[27, 33]	Rectal	2	0.846 (0.803–0.890)	0.00	0.433
**Radiologist**						
Sensitivity per-patient	[21, 31]	Colorectal	2	0.641 (0.577–0.702)	70.9	0.064
Specificity per-patient	[21, 31]	Colorectal	2	0.657 (0.597–0.713)	11.1	0.289
Sensitivity per-patient	[22–24, 26]	Rectal	4	0.678 (0.628–0.726)	57.5	0.070
Specificity per-patient	[22–24, 26]	Rectal	4	0.701 (0.667–0.733)	97.8	< 0.0001
AUROC per-patient	[21, 31]	Colorectal	2	0.676 (0.627–0.725)	58.4	0.121
AUROC per-patient	[22–24, 26, 30]	Rectal	5	0.688 (0.603 to 0.772)	93.4	< 0.0001

AUROC, area under the receiver operating characteristic; CI, confidence interval

## Discussion

To our knowledge, this is the first systematic review and meta-analysis of deep learning and radiomics performance in the assessment of lymph node metastasis in rectal and CRC patients. The results demonstrate a very high AUROC of 0.917 (95%CI, 0.882–0.952) when a deep learning model is used as a diagnostic tool compared with a radiomics model (AUROC 0.808, 95%CI 0.739–0.876). The diagnostic performance of both deep learning and radiomics models surpassed that of the radiologist assessment with an AUROC of 0.688 (95%CI, 0.603 to 0.772).

A number of research studies have already suggested AI has the potential to transform the healthcare sector particularly in areas where image recognition can be applied [35–37]. In terms of colorectal diseases, AI has been applied to colonic polyps, adenomas, colorectal cancer, ulcerative colitis and intestinal motility disorders [38–41]. Owing to the rapid development of AI technology, AI is bound to continually play an important role in the field of colorectal diagnosis and treatment [42]. Furthermore, the increase in computing power paired with the availability of large imaging databases offer the opportunity to develop more accurate AI algorithms.(10) At present, applications of deep learning to medical imaging are in vogue. However, deep learning models have several drawbacks, including variability in the images, large sample size, poor generalization and extensive computing resources. These models tend to rely on superficial data patterns and often fail when external factors such as different imaging acquisition parameters and types of scanners cause a distribution shift [43].

In this review, most studies used radiomics (*n* = 12), rather than deep learning methodology (*n* = 5) largely owing to deep learning technology being more recent, but also because it requires specific expertise. This limits the ability to draw definitive comparisons between the two AI models as one is somewhat over-represented in the data. Additionally, most studies were retrospective in design, making them prone to confounding and selection bias. Several studies focused on the technical aspects of the algorithm and did not address key limitations such as input variation, absence of clinical information (age, tumour site, patient history) and potential data overfitting often caused by noise in the data, overcomplicated models, and small sample sizes. Another issue, particularly common in deep learning studies, is the failure to report contingency tables or sufficient detail to enable reconstruction. We had to exclude five (29%) studies from the meta-analysis due to incomplete data. Most studies were conducted at a single-center and used internal verification or resampling methods (cross validation). Internal validation, however, tends to overestimate the AUROC due to the model’s lack of generalizability, limiting the integration of AI models into the clinical setting [44]. Therefore, external validation prediction models using images from different hospitals are required to create reliable estimates on the level of performance at other sites [45]. The number of studies diagnosing lymph node metastasis on a per-nodal basis in this meta-analysis is small. This is understandable, given that lymph node metastasis is staged on a per-patient basis in the clinical setting. Interestingly, five studies on rectal cancer extracted radiomics features from CT despite MRI being the gold standard imaging modality for lymph node detection in clinical practice.

This meta-analysis has some limitations that merit consideration. Firstly, a relatively small number of deep learning studies were available for inclusion. This, along with the heterogeneity seen in radiomics studies, means that the summary estimates of AUROCs have to be interpreted with caution. Secondly, because of incomplete reporting of results by several studies, estimates of diagnostic performance were calculated using limited data. Thirdly, given the majority of the included studies originate from China, there is a potential for geographical bias. Lastly, the wide range of scanner types, imaging protocols, and criteria for lymph node metastasis used may have affected accuracy of results. Results for radiomics and the radiologist assessment were highly heterogenous, which may be attributed to the different imaging modalities and small sample sizes. In the future, diagnostic AI models will have to be rigorously evaluated on their clinical benefit in comparison to current standard of care, as not all are suitable for clinical practice. Therefore, studies comparing AI with the clinicians’ performance are most valuable and are more likely to ensure safe and effective implementation of AI technology into daily practice [46, 47].

## Conclusion

AI models have the potential to predict lymph node metastasis more accurately on a per-patient basis in colorectal cancer than the radiologists’ assessment, however, radiomics studies are heterogeneous and deep learning studies are scarce. With further development and refinement, AI models capable of accurately predicting nodal stage may represent a significant advance in pre-operative staging of colorectal cancer to better inform clinician and patient.

## Supplementary Information

**Additional file 1: Table S1.** Search Strategy. **Table S2.** Diagnostic accuracy measures. **Table S3.** Selected characteristics of included studies. **Table S3.** Quality assessment of studies included in systematic review, according to the Quality Assessment of Diagnostic Accuracy Studies-2 (QUADAS-2) Tool adapted with signalling questions by Sollini et al. **Figure S1.** Publication bias presentation using funnel plot of included studies.

## Data Availability

All data generated or analysed during this study are included in this published article and supplementary material.

## Abbreviations

CRC: Colorectal cancer
MRI: Magnetic resonance imaging
CT: Computer tomography
AI: Artificial intelligence
QUADAS-2: Quality Assessment of Diagnostic Accuracy Studies tool 2
AUROC: Area under the receiver operating characteristic curve
