# Supplementary material for: Artificial intelligence for pre-operative lymph node staging in colorectal cancer: a systematic review and meta-analysis
Source: BMC Cancer. 2021 Sep 26;21:1058. doi: 10.1186/s12885-021-08773-w (PMC8474828; doi:10.1186/s12885-021-08773-w)

**Additional file 1**

Supplement to: Artificial intelligence for lymph node assessment on preoperative staging in colorectal cancer: A systematic review and meta-analysis.

**Additional file 1: Table S1. Search Strategy**

| **Sources** | **Search in** | **MeSH terms** | **Limits** | **Search results** |
| --- | --- | --- | --- | --- |
| Cochrane Library | Search manager | ("Artificial intelligence" OR "deep learning" OR "convolutional neural network" OR "machine learning" OR "automatic detection" OR "radiomics" OR "radiomic") AND ("CT" OR "MRI") AND ("Lymph node" OR "lymph node metastasis") AND ("colon" OR "rectal" OR "colorectal") | None | 3 |
| PubMed, (MEDLINE) | N/A | ("Artificial intelligence" OR "deep learning" OR "convolutional neural network" OR "machine learning" OR "automatic detection" OR "radiomics" OR "radiomic") AND ("CT" OR "MRI") AND ("Lymph node" OR "lymph node metastasis") AND ("colon" OR "rectal" OR "colorectal") | Research articles, years (2010-2020) | 14 |
| EMBASE | Quick search | ('artificial intelligence'/exp OR 'artificial intelligence' OR 'deep learning'/exp OR 'deep learning' OR 'convolutional neural network'/exp OR 'convolutional neural network' OR 'machine learning'/exp OR 'machine learning' OR 'automatic detection' OR 'radiomics'/exp OR 'radiomics' OR 'radiomic') AND ('ct'/exp OR 'ct' OR 'mri'/exp OR 'mri') AND ('lymph node'/exp OR 'lymph node' OR 'lymph node metastasis'/exp OR 'lymph node metastasis') AND ('colon'/exp OR 'colon' OR 'rectal' OR 'colorectal') | None | 45 |
| IEEE Xplore Digital Library | N/A | (“Artificial intelligence” OR “machine learning” OR “deep learning” OR “convolutional neural network” OR “automatic detection” OR “computer-aided” OR “segmentation” OR “Radiomic” OR “Radiomics”) AND (“CT” OR “MRI” OR “images” OR “diagnostic imaging” OR “radiology”) AND (“Lymph node*” OR “lymph node detection”) | None | 3 |

**Additional file 1: Table S2. Diagnostic accuracy measures**

| **Measure** | **Formula** |
| --- | --- |
| Sensitivity | $\frac{TP}{P} =\frac{TP}{TP+FN}$ |
| Specificity | $\frac{TN}{N}= \frac{TN}{TN+FP}$ |
| Accuracy | $\frac{TP+TN}{P+N}= \frac{TP+TN}{TP+TN+FP+FN}$ |
| PPV | $\frac{TP}{TP+FP}$ |
| NPV | $\frac{TN}{TN+FN}$ |
| SE | $\frac{\left( Upper Limit-Lower Limit \right)}{3.92}$ |
| 95% Confidence Interval | $best estimate+/-\left( 1.96 \right)*(SE)$ |

**Additional file 1: Table S3. Selected characteristics of included studies.**

| **First Author** | **Country** | **Year** | **Study design** | **Patients (% female patients)** | **Sample size for diagnostic accuracy, n** | **Mean or Median age (SD; range), years*** | **Imaging modality** | **Type of malignancy** | **AI model (Per-patient /per-node diagnostic output)** | **Reference standard** |
| --- | --- | --- | --- | --- | --- | --- | --- | --- | --- | --- |
| Ding [12] | China | 2020 | Prospective single-center | 545 (38%) | 183 | 58.6 (12.6) | MRI | Rectal | Deep learning (per-patient) | Pathology |
| Eresen[20] | USA | 2020 | Retrospective single-center | 390 (47%) | 78 | 62.1 (±13.25) LN (+), 62.56 (±14.17) LN (-) | CT | Colon | Radiomics (per-patient) | Pathology |
| Li[21] | China | 2020 | Prospective single-center | 766 (45%) | 308 | 59.0 (±12.03;19-87) | CT | Colorectal | Radiomics (per-patient) | Pathology |
| Yang[22] | China | 2020 | Retrospective single-center | 139 (35%) | 41 | 64 (34-86) | MRI | Rectal | Radiomics (per-patient) | Pathology |
| Nakanishi[23] | Japan | 2020 | Retrospective  Multi-center | 247 (34%) | 72 | 61 (51.3–72.8) | CT | Rectal | Radiomics (per-patient) | Pathology |
| Zhou[24] | China | 2020 | Retrospective  Single-center | 391 (29%) | 130 | 53.7 ± 11.7 | MRI | Rectal | Radiomics (per-patient) | Pathology |
| Glaser[25] | Australia | 2020 | Retrospective  Single-center | 123 | 23 | - | CT | Colon | Deep learning (per-patient) | Pathology |
| Meng [26] | China | 2019 | Retrospective  Single-center | 345 (38%) | 148 | 61.1 (±12.4) | MRI | Rectal | Radiomics (per-patient) | Pathology |
| Wang[11] | China | 2019 | Retrospective single-center | 107 | - | - | CT | Rectal | Deep learning (per-patient) | - |
| Zhu[27] | China | 2019 | Retrospective  Single-center | 215 (39%) | 72 | 58.6 (±10.3) | MRI | Rectal | Radiomics (per-node) | Pathology |
| Lu[28] | China | 2018 | Prospective multi-center | 765 | 414 | - | MRI | Rectal | Deep learning (per-node) | Pathology |
| Li[29] | China | 2018 | Retrospective single center | 619 | - | - | MRI | Colorectal | Deep learning (per-node) | Radiology |
| Chen[30] | China | 2018 | Prospective  Single-center | 115 (43%) | 33 | 57 (± 14;30–79) LN (+), 62 (± 14;29–85) | ERUS, CT, SWE | Rectal | Radiomics (per-patient) | Pathology |
| Huang[31] | China | 2016 | Retrospective  Single-center | 326 (35%) | 200 | 61.2 (±13.9), 60.0 (±13.5) LN (+), 64.9 (±11.8) LN (-) | CT | Colorectal | Radiomics (per-patient) | Pathology |
| Cai[32] | China | 2012 | Prospective  Single-center | 228 (39%) | Avg of leave-one-out CV | 58 (19-86) | CT | Rectal | Radiomics (per-node) | Pathology |
| Tse[34] | UK | 2012 | Retrospective  Multi-center | 17 | Avg of leave-one-out CV | - | MRI | Rectal | Radiomics (per-node) | Pathology |
| Cui[33] | China | 2011 | Prospective  Single-center | 228 | Avg of leave-one-out CV | - | CT | Rectal | Radiomics (per-node) | Pathology |

AI, artificial intelligence; Avg, average; CV, cross validation

**Additional file 1: Table S3. Quality assessment of studies included in systematic review, according to the Quality Assessment of Diagnostic Accuracy Studies-2 (QUADAS-2) Tool adapted with signalling questions by Sollini et al.**

(Sollini M, Antunovic L, Chiti A, Kirienko M: Towards clinical application of image mining: a systematic review on artificial intelligence and radiomics. Eur J Nucl Med Mol Imaging 2019, 46(13):2656-2672.)

| Source | RISK OF BIAS | | | | | | | | APPLICABILITY CONCERNS | | |
| --- | --- | --- | --- | --- | --- | --- | --- | --- | --- | --- | --- |
|  | PATIENT SELECTION | | | INDEX TEST | | | REFERENCE STANDARD | FLOW AND TIMING | PATIENT SELECTION | INDEX TEST | REFERENCE STANDARD |
|  | Was the statistical management adequate? | Were the inclusion/exclusion criteria specified? | Was the type of study (retrospective or prospective) specified? | Were the imaging acquisition protocol and the segmentation method(s) detailed? | Was the image  processing approach detailed? | Was the validation independent (i.e., no internal)? | Was the reference  standard adequate? | Was there an  appropriate interval between index test  and reference standard? |  |  |  |
| Ding et al 2020 [12] | yes | yes | yes | yes | yes | no | yes | unclear | yes | yes | yes |
| Eresen et al 2020 [20] | yes | yes | yes | yes | yes | no | yes | unclear | yes | yes | yes |
| Li et al 2020 [21] | yes | yes | yes | yes | yes | no | yes | unclear | yes | yes | yes |
| Yang et al 2020 [22] | yes | yes | yes | yes | yes | no | yes | unclear | yes | yes | yes |
| Nakanishi et al 2020[23] | yes | yes | yes | yes | yes | yes | yes | unclear | yes | yes | yes |
| Zhou et al 2020 [24] | yes | yes | yes | yes | yes | no | yes | unclear | yes | yes | yes |
| Glaser et al 2020 [25] | yes | no | yes | yes | yes | no | yes | unclear | yes | yes | yes |
| Meng et al 2019 [26] | yes | yes | yes | yes | yes | no | yes | unclear | yes | yes | yes |
| Wang et al 2019 [11] | yes | no | yes | yes | no | no | unclear | unclear | yes | yes | unclear |
| Zhu et al 2019 [27] | yes | yes | yes | yes | yes | no | yes | unclear | yes | yes | yes |
| Lu et al 2018 [28] | yes | yes | yes | yes | yes | yes | yes | unclear | yes | yes | yes |
| Li et al 2018 [29] | yes | yes | yes | yes | yes | no | no | unclear | yes | yes | yes |
| Chen et al 2018[30] | yes | yes | yes | yes | yes | no | yes | unclear | yes | yes | yes |
| Huang et al 2016 [31] | yes | yes | yes | yes | yes | no | yes | unclear | yes | yes | yes |
| Cai et al 2012 [32] | yes | yes | yes | yes | yes | no | yes | unclear | yes | yes | yes |
| Tse et al 2012 [34] | yes | yes | yes | yes | yes | no | yes | unclear | yes | yes | yes |
| Cui et al 2011 [33] | yes | yes | yes | yes | yes | no | yes | unclear | yes | yes | yes |

**Additional file 1: Figure S1. Publication bias presentation using funnel plot of included studies.**


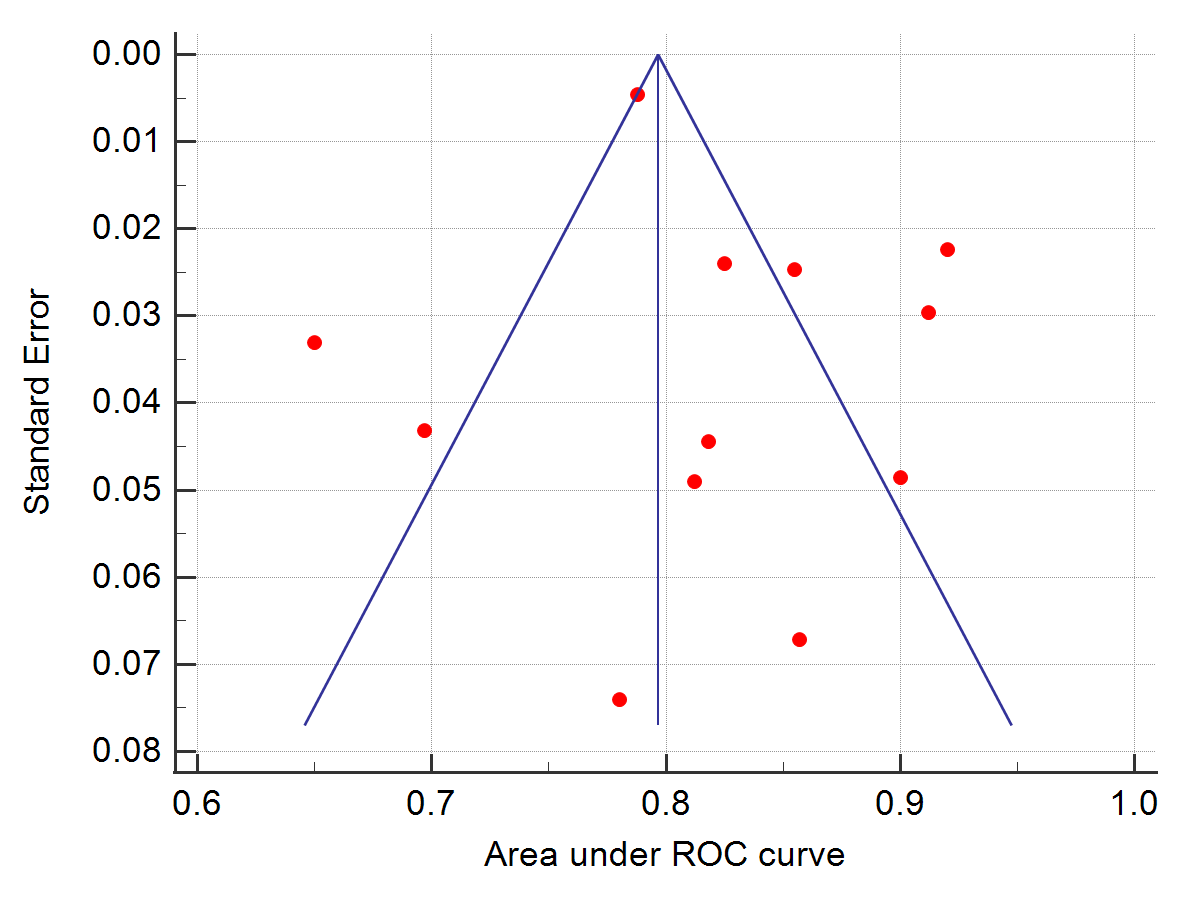

Supplement: Supplementary file 1 — Additional file 1: Table S1. Search Strategy. Table S2. Diagnostic accuracy measures. Table S3. Selected characteristics of included studies. Table S3. Quality assessment of studies included in systematic review, according to the Quality Assessment of Diagnostic Accuracy Studies-2 (QUADAS-2) Tool adapted with signalling questions by Sollini et al. Figure S1. Publication bias presentation using funnel plot of included studies. [file 12885_2021_8773_MOESM1_ESM.docx]
